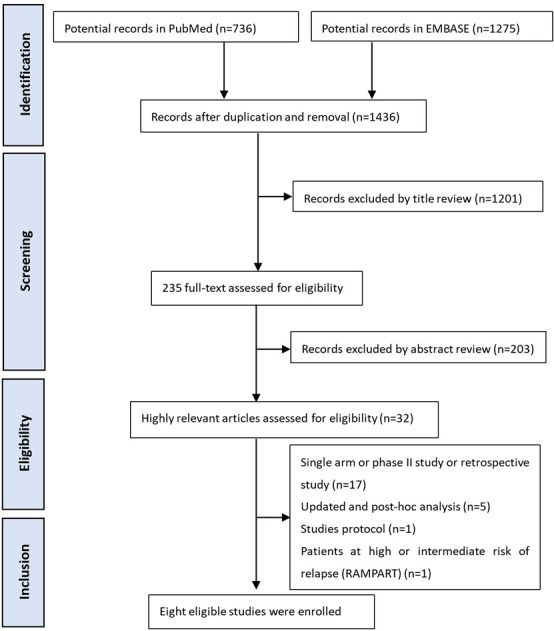# Adjuvant therapy in renal cell carcinoma: Tyrosine kinase inhibitor versus immune checkpoint inhibitor

**DOI:** 10.1097/MD.0000000000038329

**Published:** 2024-05-31

**Authors:** Qingbo Zhou, Jianjiang Liu, Shaoqin Xie

**Affiliations:** a Internal Medicine Department, Shaoxing Yuecheng People’s Hospital, Shaoxing City, Zhejiang Province, China; b Department of Radiotherapy, Shaoxing People’s Hospital, Shaoxing, Zhejiang, China; c Department of Urology, Shaoxing People’s Hospital, Shaoxing, Zhejiang, China.

**Keywords:** adjuvant therapy, immune checkpoint inhibitor, IPD, renal cell carcinoma, tyrosine kinase inhibitor

## Abstract

**Background:**

To date, no meta-analysis has been conducted to compare the effectiveness and safety of adjuvant tyrosine kinase inhibitors (TKIs) and adjuvant immunotherapies (IMTs) in renal cell carcinoma (RCC) patients using reconstructed individual patient data (IPD). This study aims to fill that gap by assessing the efficacy and safety profiles of these treatments in such patients.

**Methods:**

This study employed a systematic approach for identifying relevant literature from the PubMed and EMBASE databases. We included articles published in English from the inception of these databases until November 11, 2023, focusing specifically on appropriate phase III randomized controlled trials (RCTs). To reconstruct survival curves, we utilized a semiautomated tool, WebPlotDigitizer, in conjunction with a novel shiny application integrated with R software. For adverse events (AEs), the summary measures were incidences, expressed as a 95% confidence interval (CI), calculated using a random-effects model with a logit transformation.

**Results:**

The analysis included 8 RCTs with a total of 9119 patients. Compared to adjuvant TKIs, adjuvant IMTs showed a similar disease-free survival (DFS) (hazard ratio [HR] 1.03, 95% CI [0.98–1.09], *P* = .281). However, the overall survival (OS) rates between the 2 groups couldn’t be directly compared due to unmatched control groups in the IMT and TKI studies. Against placebo, adjuvant IMTs demonstrated superior DFS (HR 0.82, 95% CI [0.71–0.94], *P* = .004) but comparable OS (HR 0.79, 95% CI [0.59–1.06], *P* = .120). Against placebo, adjuvant TKIs showed superior DFS (HR 0.85, 95% CI [0.79–0.92], *P* < .0001) and marginally better OS (HR 0.89, 95% CI [0.80–0.996], *P* = .042). Regarding severe AEs and discontinuation rates due to AEs, adjuvant IMTs had a significantly lower incidence of severe AEs (25% [320/1282] vs 59% [2192/3716], odds ratio [OR] 0.23, 95% CI [0.20–0.27], *P* < .0001) and a markedly better discontinuation rate (39% [499/1282] vs 52% [2068/4018], OR 0.60, 95% CI [0.53–0.68], *P* < .0001) compared to TKIs.

**Conclusion:**

This paper presents a thorough analysis of DFS, OS, and treatment-related AEs across various groups in RCC patients, offering a valuable resource for clinicians in everyday practice. Our findings indicate that while adjuvant IMTs and adjuvant TKIs demonstrate similar DFS, IMTs are notably superior in terms of safety and compliance.

## 1. Introduction

The 2020 Global Cancer Statistics report indicates that renal cell carcinoma (RCC) ranks as the 14th most common cancer worldwide, accounting for 2.2% of all cancer diagnoses (431,288 cases) and 1.8% of cancer-related deaths (179,368 cases).^[1]^ The 5-year survival rate stands at 91% for early-stage RCC, but drops to 74% for locally advanced cases and plummets to 17% for metastatic disease.^[2]^ For patients eligible for surgery, the primary treatments for localized RCC include radical nephrectomy and partial nephrectomy, with other options like active surveillance or ablative techniques being viable for certain cases.^[3]^ However, despite complete resection, the 2-year recurrence rate for patients with high-risk localized RCC often surpasses 50%, and merely a third of these patients stay metastasis-free over 3 years.^[4]^ Consequently, this high-risk group could significantly benefit from adjuvant and neoadjuvant therapies to enhance disease-free survival (DFS) and, ultimately, overall survival (OS).

Several phase III randomized controlled trials (RCTs) have evaluated adjuvant tyrosine kinase inhibitors (TKIs) in RCC, but only the “S-TRAC trial” demonstrated a significant benefit in DFS in a meta-analysis.^[5]^ This meta-analysis also revealed that patients with localized and locally advanced RCC experienced a modest DFS improvement with adjuvant TKI therapy compared to placebo.^[5]^ Similarly, RCC is recognized as responsive to immunotherapy, and findings from some phase III trials involving immunotherapies (IMTs) have been reported. Recent meta-analyses suggest that RCC patients with positive PD-L1 expression or sarcomatoid features are more likely to benefit from adjuvant IMTs.^[6]^

Although prior meta-analyses have indicated that both adjuvant TKIs and IMTs may improve DFS in patients with RCC, no studies have yet directly compared the efficacy of these 2 strategies. Consequently, we have utilized reconstructed individual patient data (IPD) to indirectly compare the effectiveness of these treatment approaches, while also providing a comprehensive summary of the serious adverse events (AEs) associated with these therapies.

## 2. Methods

This study adhered to the Preferred Reporting Items for Systematic Reviews and Meta-Analyses statement, and the protocol is registered with International Prospective Register of Systematic Reviews (CRD42024494438).

### 2.1. Literature review

PubMed and EMBASE databases were searched from their inception to Nov 11, 2023, using specific search terms involving “Immunotherapy or PD-1 or programmed death receptor 1 or PD-L1 or programmed death-ligand 1 or immune checkpoint inhibitor or tremelimumab or nivolumab or pembrolizumab or atezolizumab or durvalumab or adebrelimab or avelumab or tislelizumab or ipilimumab or TKI or Tyrosine Kinase or Sunitinib or sorafenib or Pazopanib or Axitinib” in title or abstract, and “kidney cancer or RCC or RCC” in title or abstract, and “localized or postnephrectomy or adjuvant” in title or abstract. Only English language studies were reviewed, and additional relevant studies were identified from the references of the reviewed publications (see Table S1, Supplemental Digital Content, http://links.lww.com/MD/M666, which illustrates the search strategy and results in English from their inception to November 11, 2023).

### 2.2. Study selection

For inclusion in this study: All patients included were diagnosed with localized RCC; Phase III RCTs of TKIs or IMTs versus placebo in the adjuvant treatment of RCC were included in this study; Quantitative data on DFS, OS or treatment-related AEs could be obtained.

The exclusion criteria were as follows: Duplicate publications from the literature; Data that could not be extracted from the literature; Non-RCTs or phase II RCTs; the intervention drugs are not TKIs or IMTs;

The article was independently screened and scrutinized by Qingbo Zhou and Jianjiang Liu, and survival curve reconstruction was independently performed by Qingbo Zhou and Jianjiang Liu. Any disagreements were resolved through consensus.

### 2.3. Data extraction

We extracted various data from each study, including study name, year of publication, treatment protocol, number of patients, types of drugs, median age, and various other factors (e.g. the proportion of males, histologic subtype, stage for the Intervention arm, the follow-up time, the proportion of radical nephrectomy and partial nephrectomy, the discontinuation rate, the DFS rate every year, the median DFS rate every, the HR [95% CI] for DFS, etc).

### 2.4. Statistical methods

The study aimed to compare the efficacy of different treatments for RCC patients using TKIs, IMTs, and placebos. The primary outcome was the comparison of reconstructed DFS curves and the summary of specific values of DFS rates every year between groups. Secondary outcomes were the summary of rates and 95% CIs of specific severe toxicities in the TKIs and IMTs groups, comparison of OS and any severe AEs between the TKIs and IMTs groups.

Raw data coordinates were extracted using a semiautomatic tool called WebPlotDigitizer (https://apps.automeris.io/wpd/). The IPD was reconstructed using a new shiny application (https://www.trialdesign.org/one-page-shell.html#IPDfromKM) developed by Liu et al.^[7]^ The final reconstructed survival curve was plotted in R (version 4.0.3). The summary measures for AEs were incidences (95% CI). The incidences and 95% CI were calculated by logit-transformed random-effects models, and the relevant codes were published in Nyaga et al.^[8]^ Forest plots of the pooled HRs for efficacy indicators, the incidences of AEs, and the subgroup analyses were generated in STATA 14.0 (Stata Corporation, College Station, TX, USA). An α of 0.05 was considered statistically significant, and all tests were 2-sided.

## 3. Results

### 3.1. Study selection and patient characteristics

From an initial review of 2011 records across 2 databases, we selected 32 records for inclusion after screening titles and abstracts (see Figure S1, Supplemental Digital Content, http://links.lww.com/MD/M667, which illustrates the flow chart of literature search). Ultimately, our analysis incorporated 3 IMT-related^[9–12]^ and 5 TKI-related phase III RCTs.^[13–18]^ The characteristics of these included trials are detailed in Table 1. Notably, the TKI groups had a longer median follow-up time compared to the IMT groups. Additionally, Table 2 summarizes the time-specific DFS rates and the median DFS across the various studies.

**Table 1:** Characteristics of the included trials of the present study.

Study, year	Arm	ICIs	N	Median age	Males (%)	Smoke (%)	ECOG1 (%)	BM (%)	FU (M)
KEYNOTE-604, 2020^[10]^	Pembrolizumab + CT	PD-1	228	64	66.7	96.5	73.7	14.5	NR
	CT		225	65	63.1	96.4	75.1	9.8	NR
ASTRUM-005, 2022^[9]^	Serplulimab + CT	PD-1	389	63	81.5	79.2	81.7	12.9	12.5
	CT		196	62	83.7	82.1	83.7	14.3	12.3
CAPSTONE-1, 2022^[8]^	Adebrelimab + CT	PD-L1	230	62	80	78	86	5	14.4
	CT		232	62	81	77	87	5	12.8
IMpower133, 2021^[6]^	Atezolizumab + CT	PD-L1	201	64	64.2	95.5	63.7	8.5	22.9
	CT		202	64	65.3	98.5	66.8	8.9	22.9
CASPIAN, 2021^[7]^	Durvalumab + CT	PD-L1	268	62	71	92	63	10	25.1
	CT		269	63	68	94	67	10	25.1

BM = brain metastasis, CT = chemotherapy, ECOG = Eastern cooperative oncology group, FU = follow-up, M = median, ICIs = immune checkpoint inhibitors, N = number, NR = not reported, PD-1 = programmed cell death 1 inhibitors, PD-L1 = programmed cell death-ligand 1 inhibitors.

**Table 2:** Progression-free survival (PFS), overall survival (OS) and objective response rate (ORR) outcomes and adjusted hazard ratios (HR) of included studies.

Study, yr	Arm	6-moPFS (%)	12-moPFS (%)	18-moPFS (%)	24-moPFS (%)	Median PFS	Adjusted HR (95% CI) for PFS	6-moOS (%)	12-moOS (%)	18-moOS (%)	24-moOS (%)	Median OS	Adjusted HR (95% CI) for OS	ORR (%)	RR (95%CI) for ORR
KEYNOTE-604,2020^[10]^	Pembrolizumab + CT	34.10	13.60	6.86	NR	4.5	0.75 (0.61–0.91)	77.15	45.10	29.49	22.50	10.8	0.80 (0.64–0.98)	71	0.88 (0.76–1.00)
	CT	23.80	3.10	0.98	NR	4.3	Ref	74.80	39.60	22.07	11.20	9.7	Ref	62	Ref
ASTRUM-005, 2022^[9]^	Serplulimab + CT	47.55	23.77	NR	NR	5.7	0.48 (0.38–0.59)	88.95	60.87	46.01	NR	15.4	0.63 (0.49–0.82)	80.2	0.88 (0.79–0.97)
	CT	19.81	6.23	NR	NR	4.3	Ref	77.90	47.83	29.89	NR	10.9	Ref	70.4	Ref
CAPSTONE-1, 2022^[8]^	Adebrelimab + CT	49.47	19.79	16.84	14.74	5.8	0.67 (0.54–0.83)	87.83	63.27	42.48	31.42	15.3	0.72 (0.58–0.90)	70.4	0.93 (0.83–1.06)
	CT	37.89	6.11	5.26	2.95	5.6	Ref	90.71	51.99	27.43	17.26	12.8	Ref	65.9	Ref
IMpower133, 2021^[6]^	Atezolizumab + CT	31.32	12.75	9.62	0	5.2	0.77 (0.63–0.95)	85.80	50.71	32.86	21.70	12.3	0.76 (0.60–0.95)	60	1.06 (0.92–1.25)
	CT	22.37	5.37	≤1.79	NR	4.3	Ref	83.98	39.76	21.10	17.04	10.3	Ref	64	Ref
CASPIAN, 2021^[7]^	Durvalumab + CT	45.45	17.87	13.83	11.07	5.1	0.80 (0.66–0.96)	80.43	52.77	32.02	22.13	12.9	0.71 (0.60–0.86)	68	0.85 (0.75–0.97)
	CT	46.05	5.14	3.36	2.96	5.4	Ref	79.84	39.33	24.31	14.43	10.5	Ref	58	Ref

Survival data was extracted from Kaplan–Meier survival curves by data extraction software.

CI = confidence interval, CT = chemotherapy, Ref = reference, RR = risk ratio.

### 3.2. Comparisons for each study in terms of disease-free survival and overall survival

**Figure 1.**
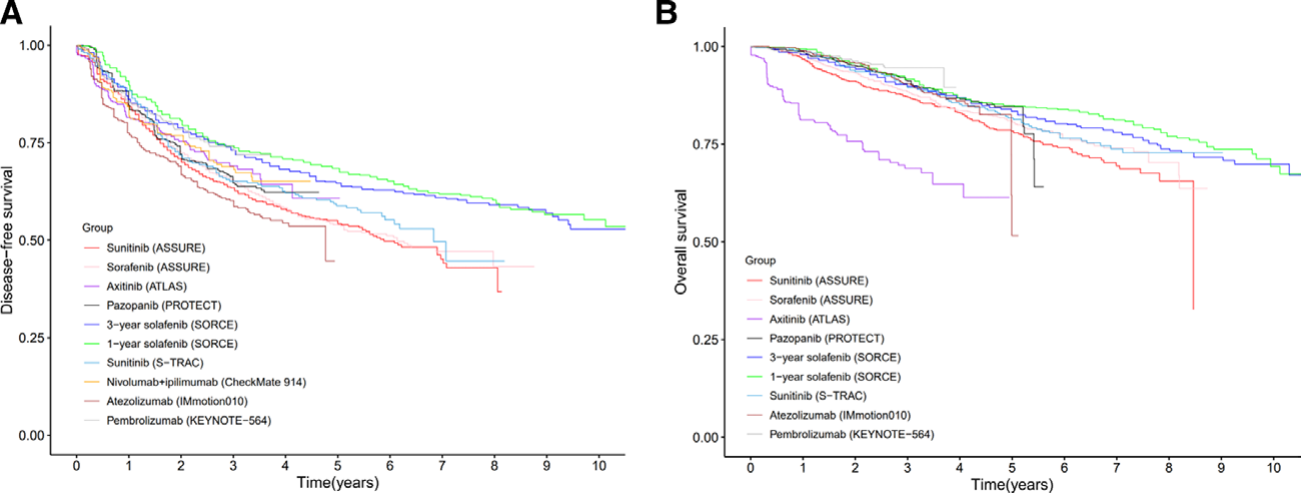
Reconstructed Kaplan–Meier survival curves of DFS (A) and overall survival (OS) (B) in different studies.

As illustrated in Figure 1A and outlined in Table 2, patients treated with 1- or 3-year courses of sorafenib in the SORCE study, as well as those receiving 1-year pembrolizumab in KEYNOTE-564, demonstrated superior DFS. In contrast, patients on a 1-year course of atezolizumab in the IMmotion010 study reported the lowest DFS. Additionally, Figure 1B shows that while patients treated with 1 to 3 years of axitinib in the ATLAS study had moderate DFS, their OS was the least favorable compared to other studies.

### 3.3. Adjuvant immunotherapies versus tyrosine kinase inhibitor in Terms of disease-free survival and overall survival

**Figure 2.**
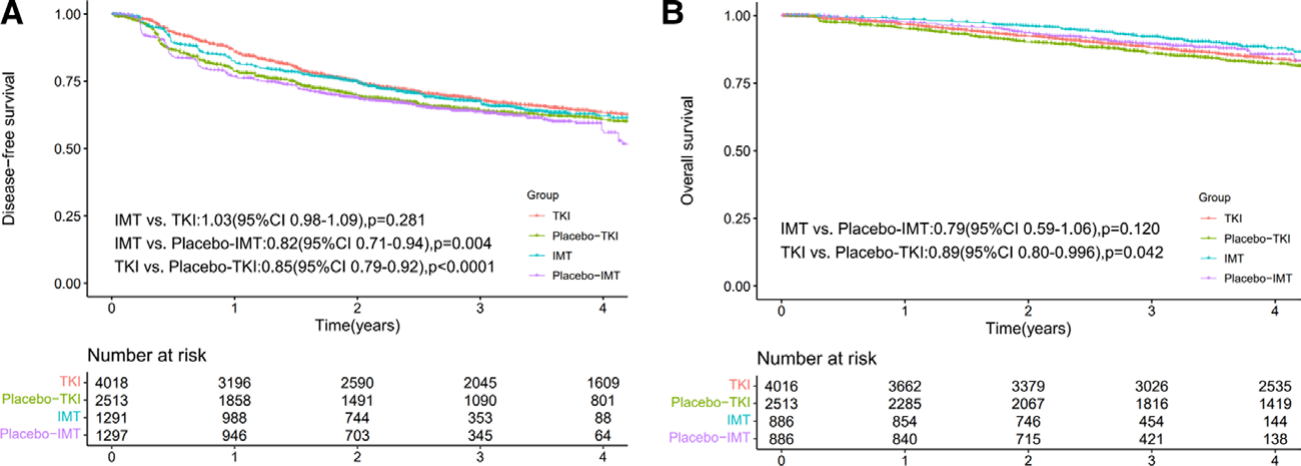
Reconstructed Kaplan–Meier survival curves of DFS (A) and OS (B) in the TKI, Placebo-TKI, IMT and Placebo-IMTI groups.

In terms of DFS, patients treated with adjuvant IMTs showed comparable results (HR, 1.03 [95% CI 0.98–1.09], *P* = .281) to those receiving adjuvant TKIs, as depicted in Figure 2A. Both the IMT-placebo and TKI-placebo groups were closely matched. However, the OS rates between the 2 groups can’t be directly compared due to the mismatch in the control groups, that is, the IMT-placebo and TKI-placebo groups (Fig. 2B). Compared to placebo, adjuvant IMTs led to superior DFS (HR, 0.82 [95% CI 0.71–0.94], *P* = .004) but similar OS (HR, 0.79 [95% CI 0.59–1.06], *P* = .120). In contrast, adjuvant TKIs not only demonstrated significantly better DFS (HR, 0.85 [95% CI 0.79–0.92], *P* < .0001) but also a marginally improved OS (HR, 0.89 [95% CI 0.80–0.996], *P* = .042) as shown in Figures 2A and 2B.

### 3.4. Comparisons for different periods in terms of disease-free survival and overall survival

**Figure 3.**
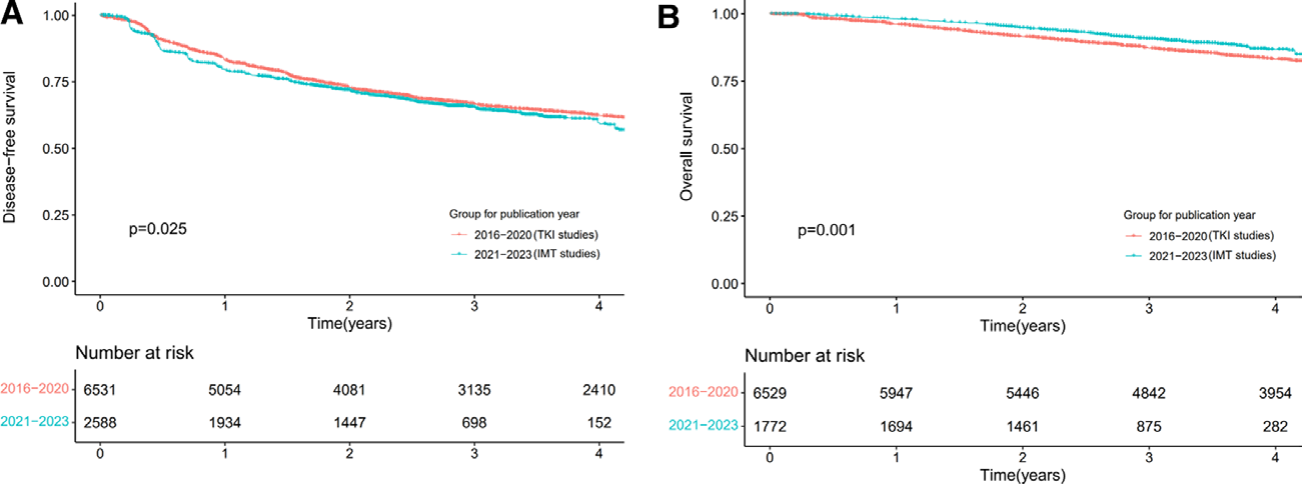
Reconstructed Kaplan–Meier survival curves of DFS (A) and OS (B) between different publication years.

Furthermore, we conducted a subgroup analysis for different time periods, including patients from the placebo group. As illustrated in Figures 3A and 3B, the studies conducted between 2016 and 2020 were exclusively TKI-related, whereas those from 2021 to 2023 focused on IMTs. Despite the *P* value being <.05, the DFS outcomes for both groups were quite similar. Regarding OS, the group from 2021 to 2023 showed a slight improvement over the 2016 to 2020 group (*P* = .001).

### 3.5. Safety and compliance of the patients treated with adjuvant immunotherapies and tyrosine kinase inhibitor

**Figure 4.**
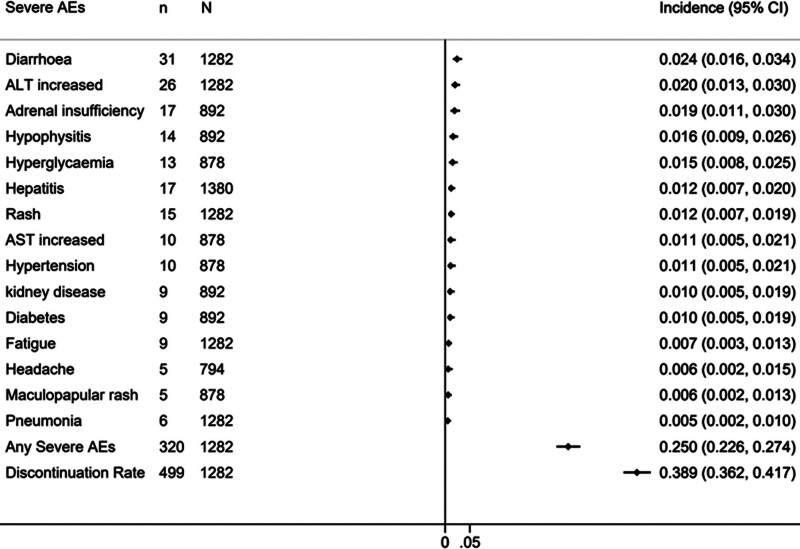
Incidences of severe AEs in the patients treated with adjuvant IMTs. Note: AEs adverse events, IMTs Immunotherapies, ALT alanine aminotransferase, AST aspartate aminotransferase; “n” means the number of patients with severe AEs, “N” means the total number of patients.

**Figure 5.**
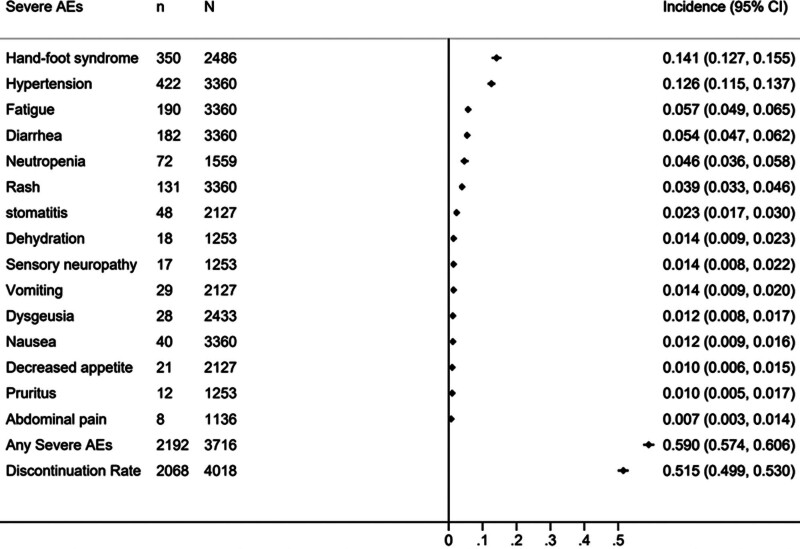
Incidences of severe AEs in the patients treated with adjuvant TKIs. Note: TKIs Tyrosine kinase inhibitors, AEs adverse events, “n” means the number of patients with severe AEs, “N” means the total number of patients.

The profiles of severe AEs for the adjuvant IMT and TKI groups are detailed in Figures 4 and 5. It’s important to note that the serious AEs listed in Figures 4 and 5 are not exhaustive; many AEs were not summarized due to their low incidence or rarity. In the adjuvant IMT group, the most frequent severe AEs were diarrhea (2.4%, 95% CI [1.6%–3.4%]), increased alanine aminotransferase (ALT) (2%, 95% CI [1.3%–3%]), and adrenal insufficiency (1.9%, 95% CI [1.1%–3%]). The incidence of any severe AE and the discontinuation rate due to AEs were 25% (95% CI, 23%–27%) and 39% (95% CI, 36%–42%), respectively. In the adjuvant TKI group, the most common severe AEs included Hand-foot syndrome (14.1%, 95% CI [12.7%–15.5%]), hypertension (12.6%, 95% CI [11.5%–13.7%]), and fatigue (5.7%, 95% CI [4.9%–6.5%]), with the incidence of any severe AE and the discontinuation rate at 59% (95% CI, 57%–61%) and 52% (95% CI, 50%–53%), respectively. When comparing severe AEs and discontinuation rates due to AEs, adjuvant IMTs had a significantly lower incidence of serious AEs (25% [320/1282] vs 59% [2192/3716], OR: 0.23, 95% CI 0.20–0.27, *P* < .0001) and a notably better discontinuation rate (39% [499/1282] vs 52% [2068/4018], OR: 0.60, 95% CI 0.53–0.68, *P* < .0001) compared to TKIs.

## 4. Discussion

RCC is essentially a metabolic disease characterized by reprogramming of energy metabolism.^[19–22]^ In RCC, this metabolic reprogramming encompasses several pathways, including enhanced glycolysis, impaired mitochondrial function, and alterations in lipid metabolism. Despite the presence of oxygen, RCC cells prefer glycolysis for adenosine triphosphate production, a phenomenon known as “aerobic glycolysis.”^[20,23–25]^ Furthermore, mitochondrial functions are often inhibited in RCC, which forces cells to rely more on glycolysis rather than oxidative phosphorylation for energy production.^[26]^ Additionally, studies have indicated changes in lipid metabolism in RCC cells, involving both synthesis and degradation, which may relate to cell membrane assembly and maintenance or the regulation of signaling pathways.^[23,27–29]^ These characteristics of metabolic reprogramming are crucial for understanding the biology of RCC and can influence how tumors respond to treatment. Moreover, various intracellular signaling pathways can affect the choice and operation of metabolic pathways, with the mammalian Target of Rapamycin signaling pathway being notably significant. Aberrant activation of the mammalian Target of Rapamycin pathway in RCC has been closely linked to tumor development and progression,^[30–32]^ making inhibition of this pathway a strategic approach in RCC treatment.

RCC is also 1 of the tumors with the most severe immune infiltration, characterized by the presence of numerous immune cells such as T cells, natural killer cells, and antigen-presenting cells around the tumor.^[33–35]^ This immune infiltration could be triggered by tumor-derived antigens that provoke an immune response, although the tumor might also employ various mechanisms to evade immune attack, such as inhibiting immune cell function or altering the tumor microenvironment to suppress immune responses. Recent studies suggest that specific metabolic pathways play a significant role in regulating the angiogenesis and inflammatory characteristics of RCC.^[36,37]^ Activation of these metabolic pathways could influence tumor immune characteristics in several ways. For instance, it might alter the supply of nutrients and metabolites in the tumor microenvironment, thereby affecting the functionality and quantity of immune cells.^[38,39]^ Additionally, activation of metabolic pathways could directly impact the immune characteristics of tumor cells, such as by modulating antigen presentation and processing pathways.^[38,40,41]^ In such cases, immunosuppressants might be an effective treatment strategy, especially for tumors with significant immune infiltration. Furthermore, the features of the tumor microenvironment heavily affect disease biology and may influence responses to systemic therapy.^[42–45]^

This meta-analysis is, to the best of our knowledge, the first to reconstruct and analyze DFS and OS curves, evaluating the efficacy of adjuvant IMTs and TKIs in RCC patients. It also represents the first comprehensive investigation into the serious AEs commonly associated with these 2 treatment strategies, comparing the incidence of severe AEs and the discontinuation rates due to AEs. Our study demonstrates that both adjuvant IMT and TKI therapies offer a slight benefit in DFS for patients undergoing nephrectomy. In our study, the HRs (95% CI) for DFS and OS in the TKI group were 0.85 (95% CI 0.79–0.92) (DFS: TKIs vs placebos, *P* < .0001) and 0.89 (95% CI 0.80–0.996) (OS: TKIs vs placebos, *P* = .042), respectively. These findings align closely with those from Laukhtina et al,^[5]^ who reported an HR of 0.88 (95% CI 0.81–0.96) for DFS (TKIs vs placebos, *P* = .004) and a slightly different HR of 0.93 (95% CI 0.83–1.04) for OS (TKIs vs placebos, *P* = .23). In our study, the HR for DFS in the IMT group was 0.82 (95% CI 0.71–0.94) (DFS: IMTs vs placebos, *P* = .004), which slightly varies from Riveros et al^[6]^ who reported an HR of 0.81 (95% CI 0.63–1.05) (DFS: IMTs vs placebos, *P* = .11). It is important to note that IPD meta-analysis and conventional meta-analysis are distinct methods. Nevertheless, our HRs and 95% CIs closely match those in other studies, reinforcing the validity of this method for comparing the efficacy of adjuvant TKIs and IMTs in RCC patients. Furthermore, for a fair comparison between IMT and TKI groups, the placebo groups in each setting must be similar in terms of DFS or OS. Significant differences in the control group results could introduce bias, potentially rendering the comparison unreliable. Fortunately, our study indicates a high overlap of DFS curves between the placebo-TKIs and placebo-IMTs groups (Fig. 2A), fulfilling a critical condition for comparing DFS in adjuvant TKIs and IMTs. However, due to the mismatch in control groups, we cannot conclusively state that adjuvant IMTs are superior to TKIs in improving OS for RCC patients, despite the higher survival curve in the adjuvant IMTs group (Fig. 2B).

Toxicity profiles are crucial considerations for informed patient consent, and vigilant monitoring with early symptom identification is key for effective management. Thus, we’ve meticulously summarized the major serious toxicity profiles for both treatment strategies, offering valuable clinical insights. This meta-analysis revealed distinct differences in the incidence of major serious AEs between adjuvant IMTs and TKIs. In the adjuvant IMT group, the most prevalent serious AEs were diarrhea (2.4%), increased ALT (2%), and adrenal insufficiency (1.9%). Conversely, the adjuvant TKI group primarily experienced Hand-foot syndrome (14.1%), hypertension (12.6%), and fatigue (5.7%). Notably, diarrhea was more common in the TKI group (5.4%) compared to the IMT group (2.4%). Hand-foot syndrome, a TKI-specific adverse reaction, was reported in various TKI-related RCTs, including studies on sorafenib, sunitinib, and axitinib, besides Pazopanib in the PROTECT study. Furthermore, the incidences of hypertension and fatigue were markedly lower in the IMT group (1.1% and 0.7%, respectively) than in the TKI group (12.6% and 5.7%). Other concerning AEs like rash were also more prevalent with TKIs (3.9% vs 1.2%). Although no individual serious AE in the IMT group exceeded 2.5%, the total incidence of serious AEs was still high at 25%, possibly due to the broad spectrum of immune-related AEs. However, this incidence was significantly lower than in the TKI group (25% vs 59%, OR: 0.23 [95% CI 0.20–0.27], *P* < .0001). Additionally, patients on adjuvant IMTs were less likely to discontinue treatment due to AEs compared to those on TKIs (39% vs 52%, OR: 0.60 [95% CI 0.53–0.68], *P* < .0001). Therefore, despite similar DFS outcomes, IMTs clearly offer superior safety and tolerability.

The indirect comparison of survival outcomes across different populations via survival curve reconstruction is a valid approach. However, the reliability of the results hinges on the high homogeneity of each included study. In this research, variations in baseline characteristics among the enrolled patients, such as intervention group stage, histological subtypes, treatment duration, and median follow-up time, could potentially influence our conclusion regarding the similarity in DFS between the 2 treatment strategies. Furthermore, factors like different data extraction software, survival reconstruction methods, the resolution of survival curves, and the researcher’s operational techniques might impact the accuracy of data recovery during this process. It’s important to remember that reconstructed IPD can never fully replicate the original data, which in turn can affect the conclusions drawn from this reconstructed IPD. Consequently, the reliability of our findings may be somewhat limited, underlining the need for further verification through relevant RCTs.

## 5. Conclusion

This paper offers a thorough analysis of DFS, OS, and treatment-related AEs among various RCC patient groups, serving as a valuable reference for clinicians in their practice. Our findings reveal that both adjuvant IMTs and TKIs slightly improve DFS compared to placebos. While these 2 adjuvant treatments demonstrate similar DFS outcomes, IMTs distinctly outperform TKIs in terms of safety and tolerability.

## Author contributions

**Conceptualization:** Qing Bo Zhou.

**Data curation:** Qing Bo Zhou, Jianjiang Liu, Shaoqin Xie.

**Formal analysis:** Qing Bo Zhou, Jianjiang Liu.

**Funding acquisition:** Qing Bo Zhou.

**Investigation:** Qing Bo Zhou, Shaoqin Xie.

**Methodology:** Qing Bo Zhou.

**Project administration:** Qing Bo Zhou.

**Resources:** Qing Bo Zhou.

**Software:** Qing Bo Zhou, Jianjiang Liu.

**Supervision:** Qing Bo Zhou, Jianjiang Liu.

**Validation:** Jianjiang Liu, Shaoqin Xie.

**Writing – original draft:** Qing Bo Zhou.

**Writing – review & editing:** Qing Bo Zhou, Shaoqin Xie.

## Supplementary Material



**Figure:**