# Supplementary material for: Adjuvant therapy in renal cell carcinoma: Tyrosine kinase inhibitor versus immune checkpoint inhibitor
Source: Medicine (Baltimore). 2024 May 31;103(22):e38329. doi: 10.1097/MD.0000000000038329 (PMC11142775; doi:10.1097/MD.0000000000038329)
Supplement: Supplementary file 1 [file medi-103-e38329-s001.docx]

Supplementary Table 1. Search strategy and results in English from their inception to Nov 11, 2023.

| Database | Search query | Results |
| --- | --- | --- |
| Pubmed | (((((((((((((((((((((((((((immunotherapy[Title/Abstract])) OR (PD-1[Title/Abstract])) OR (programmed death receptor 1[Title/Abstract])) OR (PD-L1[Title/Abstract])) OR (programmed death-ligand 1[Title/Abstract])) OR (immune checkpoint inhibitor[Title/Abstract])) OR (tremelimumab[Title/Abstract])) OR (nivolumab[Title/Abstract])) OR (pembrolizumab[Title/Abstract])) OR (atezolizumab[Title/Abstract])) OR (durvalumab[Title/Abstract])) OR (adebrelimab[Title/Abstract])) OR (avelumab [Title/Abstract])) OR (tislelizumab[Title/Abstract]))OR (ipilimumab [Title/Abstract])) OR (TKI [Title/Abstract])) OR (Tyrosine Kinase [Title/Abstract])) OR (Sunitinib [Title/Abstract])) OR (sorafenib [Title/Abstract])) OR (Pazopanib [Title/Abstract])) OR (Axitinib[Title/Abstract])) AND ((kidney cancer[Title/Abstract]) OR (Renal Cell Carcinoma [Title/Abstract]) OR (RCC [Title/Abstract]))) AND ((localized[Title/Abstract]) OR (postnephrectomy[Title/Abstract])OR (adjuvant[Title/Abstract]))) | 736 |
| Embase | ('Renal Cell Carcinoma':ab,ti OR 'kidney cancer':ab,ti OR 'RCC':ab,ti) AND (' localized':ab,ti OR 'postnephrectomy':ab,ti OR 'adjuvant':ab,ti) AND ('immunotherapy':ab,ti OR 'immune checkpoint inhibitor':ab,ti OR 'PD-1':ab,ti OR 'PD-L1':ab,ti OR ' programmed death receptor 1':ab,ti OR 'programmed death-ligand 1':ab,ti OR 'nivolumab':ab,ti OR 'pembrolizumab':ab,ti OR 'atezolizumab':ab,ti OR 'durvalumab':ab,ti OR 'avelumab':ab,ti OR 'camrelizumab':ab,ti OR 'ipilimumab':ab,ti OR 'adebrelimab':ab,ti OR 'tislelizumab':ab,ti OR 'tremelimumab':ab,ti OR 'TKI':ab,ti OR 'Tyrosine Kinase':ab,ti OR 'Sunitinib':ab,ti OR 'sorafenib':ab,ti OR 'Pazopanib':ab,ti OR 'Axitinib':ab,ti) | 1275 |
